# Temporal association between antibiotic use and resistance in *Klebsiella pneumoniae* at a tertiary care hospital

**DOI:** 10.1186/s13756-018-0373-6

**Published:** 2018-07-16

**Authors:** Sukhyun Ryu, Eili Y. Klein, Byung Chul Chun

**Affiliations:** 1 Division of Infectious Disease Control, Gyeonggi Provincial Government, Suwon, Republic of Korea; 2 Department of Epidemiology and Health Informatics, Graduate School of Public Health, Korea University, Seoul, Republic of Korea; 3 Center for Disease Dynamics, Economics & Policy, Washington, D.C USA; 4 Department of Emergency Medicine, Johns Hopkins University, Baltimore, USA; 5 Department of Preventive Medicine, Korea University College of Medicine, 73 Inchon-ro, Seongbuk-gu, Seoul, 02841 Republic of Korea

**Keywords:** β-Lactamase inhibitor, *Klebsiella pneumoniae*, Piperacillin-tazobactam, Carbapenem resistance, Cross-correlation

## Abstract

**Background:**

β-Lactam/β-lactamase inhibitors (BLBLIs) were introduced into clinical practice as an alternative to carbapenems for treating multi-drug–resistant *Klebsiella pneumoniae* infections. However, little is known about the relationship between BLBLI treatment and antimicrobial resistance. In this study, we investigated the trends and the temporal association between antibiotic use and antimicrobial resistance in *K. pneumoniae* isolates obtained between 2012 and 2016.

**Methods:**

Data regarding quarterly consumption (total number of prescriptions per quarter) of all BLBLIs, all third-generation cephalosporins, and all fluoroquinolones at a tertiary care hospital were obtained from the Korean Health Insurance Review and Assessment Service. Susceptibility data (isolation rate of antibiotic resistance per quarter) were obtained from the existing database of the same tertiary hospital. Regression analysis was used to analyze annual trends and cross-correlations to assess the temporal association on a quarterly basis between antibiotic consumption and antibiotic resistance in *K. pneumoniae*.

**Results:**

The rate of resistance to piperacillin/tazobactam in *K. pneumoniae* significantly increased over the study period (*p* < 0.01). The consumption of all BLBLIs was also found to be significantly correlated with the rate of resistance to piperacillin/tazobactam (β = 0.66; *p* < 0.01), ceftazidime (β = 0.54; *p* = 0.02), and levofloxacin (β = − 0.60; *p* = 0.01) with two-quarter lags. Furthermore, the consumption of all third-generation cephalosporins was significantly correlated with rates of *K. pneumoniae* resistance to ceftazidime (β = 0.64; *p* < 0.01) with a two-quarter lag and levofloxacin (β = 0.50; *p* = 0.03) with a quarter lag. The consumption of all fluoroquinolones correlated with resistance rates to ceftazidime (β = 0.14; *p* < 0.01) with a two-quarter lag.

**Conclusions:**

The rate of resistance to piperacillin/tazobactam in *K. pneumoniae* increased significantly over the study period and was significantly correlated with BLBLI consumption. While BLBLIs can potentially be utilized as an alternative to carbapenems, our findings reinforce concerns of resistance to these drugs. Further research is needed to understand the implications on resistance of utilizing BLBLIs as a carbapenem-sparing option.

## Background

*Klebsiella pneumoniae* is a major nosocomial pathogen that causes pneumonia, urinary tract infections, and bacteremia [1]. The widespread use of broad-spectrum cephalosporins over the last several decades has resulted in extended-spectrum β-lactamase (ESBL)–producing strains of *K. pneumoniae* becoming endemic in hospitals worldwide [2, 3]. ESBLs hydrolyze β-lactam antibiotics such as penicillins and most cephalosporins, thereby conferring resistance to these drugs [4, 5]. Thus, ESBL-producing *K. pneumoniae* are usually resistant to at least one extended-spectrum cephalosporin [6]. Delays in effective treatment result in worse outcomes including increased length of hospital stays, hospitalization costs, and mortality compared to infections caused by non-ESBL producing strains [6]. The burden caused by these strains is particularly high in Korean hospitals, where approximately 53% of *K. pneumoniae* infections are caused by ESBL-producing strains [7].

To overcome this β-lactamase–mediated resistance and to enhance the efficacy of β-lactam antibiotics, β-lactam/β-lactamase inhibitor (BLBLI) combinations such as ampicillin/sulbactam and piperacillin/tazobactams have been introduced in clinical practice [8]. Although these drugs are effective in treating infections caused by ESBL-producing organisms [8, 9], carbapenems have largely remained the treatment of choice [2, 11], as studies have reported conflicting results regarding the relative efficacy of therapy with BLBLIs compared to that with carbapenems [12]. However, the emergence of carbapenemase-producing *Enterobacteriaceae* [13, 14] has increased the need to utilize carbapenem-sparing treatment options, such as BLBLIs, for ESBL-related infections [10, 11]. However, it is now a growing concern that the increased use of BLBLIs will likely lead to increased resistance to these drugs [15, 16]. One previous molecular study supports this concern, indicating that the use of BLBLIs selects for point mutants in TEM penicillinases resistant to inhibitors [17].

Although there is a generally well-established link between overall antibiotic use and resistance within hospitals [18, 19], information is still lacking on the relationship between BLBLI consumption and antibiotic resistance in *K. pneumoniae.* In this study, we examined the temporal association between the consumption of antibiotics commonly used in hospitals, particularly BLBLIs, third-generation cephalosporins, and fluoroquinolones, and antimicrobial resistance in *K. pneumoniae.* This study aimed to increase understanding of the relationship between the use of and resistance to BLBLIs to improve decision-making and policies on the use of BLBLIs as a carbapenem-sparing option.

## Methods

### Antibiotic prescription data

The data for antibiotic utilization (total number of antibiotics prescribed) per quarter in a designated hospital was obtained from the database of the Korean Health Insurance Review and Assessment Service (KHIRA). KHIRA covers 98% of the Korean population and includes prescription data for patients from all Korean medical institutions based on financial claims [20]. The antibiotics from the database were coded according to the World Health Organization (WHO)‘s Anatomical Therapeutic Chemical (ATC) Classification [21]. Since classes of antibiotics have similar underlying mechanisms against bacteria, this classification is widely used to measure the relationship between prevalence of bacterial isolates’ resistance and the exposure to different antibiotics [22, 23]. Thus, systemic antibiotic prescriptions for all BLBLIs (ATC code: J01CR), all third-generation cephalosporins (J01DD), and all fluoroquinolones (J01MA) from the hospital were included.

### Antimicrobial resistance data

Microbial culture data were extracted from the existing database of the hospital and aggregated to generate quarterly estimates of antibiotic resistance [24]. The hospital has 2200 beds and has been designated as a Korean antimicrobial resistance monitoring focal point by the WHO since 1988 [25]. The specimens were collected from outpatient and inpatient departments. Identification and susceptibility testing were performed using an automated system (Vitek; bioMerieux) or disk diffusion tests in accordance with Clinical Laboratory Standards Institute (CLSI) criteria [24]. Piperacillin/tazobactam, ceftazidime, and levofloxacin were used for the susceptibility test, representing BLBLIs, third-generation cephalosporins, and fluoroquinolones, respectively. Duplicate isolates and intermediate susceptibility were not included in the resistance data [26–28]. The quarterly rate of piperacillin/tazobactam, ceftazidime, and levofloxacin resistance of *K. pneumoniae* isolates were obtained. The resistance rates were calculated as the number of resistant isolates divided by the number of tests in each quarter/year.

### Statistical analyses

We used regression analysis to determine the trends of annual antibiotic prescriptions and antimicrobial resistance during the study period. *P* values less than 0.05 with R-squared values greater than 0.3 were considered statistically significant [29]. Furthermore, to identify the temporal relationship between the quarterly number of antibiotics prescriptions and the quarterly isolation rate of antibiotic susceptibility, a cross-correlation function test was performed. This cross-correlation function test is widely used to identify the time lags of one time series (antibiotic prescribing) with the possible predictors of another time series (antibiotic resistance) [30]. The Box-Jenkins method was applied to fit the time series data to seasonal autoregressive moving average models [31, 32]. Stationary was evaluated using the augmented Dickey-Fuller test to determine whether differencing was required to rule out spurious correlations. The Akaike information criterion test and the portmanteau test of the residuals were used to identify the best model fit. The residuals from each time series model were used to evaluate the temporal relationship between antibiotics prescription and isolation rate of *K. pneumoniae* with antibiotic resistance [33]. All statistical analyses were performed using R version 3.2.4 (R Foundation for Statistical Computing, Vienna, Austria).

## Results

The average annual number of antibiotics prescriptions during the study period was 69,100 (range 27,371–84,128) for all BLBLIs; 53,300 (range 22,789–70,514) for all third-generation cephalosporins; and 28,536 (range 12,324–35,368) for all fluoroquinolones (Table 1). There was no significant trend in prescribing rates over the study period.

**Table 1:** Average quarterly number of antibiotic prescriptions in a tertiary hospital from 2012 to 2016

Antibiotics	Annual mean number of antibiotics prescriptions				
	2012	2013	2014	2015	2016
β-lactam/β-lactamase inhibitors	68,907 (53,899–80,221)	72,725 (63,235–84,128)	71,561 (68,880–76,537)	63,638 (27,371–80,928)	68,512 (64,378–71,719)
Third-generation cephalosporins	63,304 (60,238–70,514)	64,784 (60,468–67,074)	51,578 (44,996–54,267)	39,007 (22,789–46,250)	45,999 (44,346–47,384)
Fluoroquinolones	29,300 (26,233–33,663)	31,621 (28,920–35,297)	27,582 (21,069–35,286)	25,900 (12,324–32,129)	28,195 (19,737–35,368)

Data indicate mean number of prescriptions (Min–Max)

The yearly numbers of *K. pneumoniae* isolates from 2012 to 2016 for this analysis were 1524, 1609, 1585, and 1754. The mean rates of *K. pneumoniae* resistance to piperacillin/tazobactam, ceftazidime, and levofloxacin were 41% (range 22–49), 34% (range 14–44), and 30% (range 23–44), respectively (Table 2). There was a stable trend in resistance rates against ceftazidime and levofloxacin; however, the resistance rate against piperacillin/tazobactam significantly increased from 34% (range: 22–46) in 2012 to 46% (range: 43–49) in 2016; (*p* < 0.01).

**Table 2:** Quarterly antimicrobial resistance in *K. pneumoniae* in a tertiary hospital from 2012 to 2016

Antibiotics	Isolation rate (%) of antibiotic-resistant *Klebsiella pneumoniae*				
	2012	2013	2014	2015	2016
Piperacillin/Tazobactam	33.75 (22.00–46.00)	42.50 (40.00–45.00)	41.00 (39.00–45.00)	40.25 (30.00–46.00)	46.00 (43.00–49.00)
Ceftazidime	40.75 (38.00–44.00)	38.50 (36.00–42.00)	28.75 (26.00–31.00)	27.00 (14.00–35.00)	36.00 (33.00–38.00)
Levofloxacin	28.50 (27.00–32.00)	29.00 (27.00–31.00)	26.25 (23.00–30.00)	32.50 (27.00–44.00)	33.00 (30.00–36.00)

Data indicate mean number of prescriptions (Min–Max)

In a bivariate analysis, the lagged (two quarters) quarterly number of all BLBLI prescriptions significantly correlated with *K. pneumoniae* resistance to piperacillin/tazobactam (β = 0.66; *p* < 0.01), ceftazidime (β = 0.54; *p* = 0.02), and levofloxacin (β = − 0.60; *p* = 0.01). Similarly, the lagged quarterly number of all third-generation cephalosporin prescriptions significantly correlated with *K. pneumoniae* resistance to ceftazidime (β = 0.64; *p* < 0.01; two quarters) and levofloxacin (β = 0.50; *p* = 0.03; one quarter). The lagged quarterly number of all quinolone prescriptions correlated with *K. pneumoniae* resistance to ceftazidime (β = 0.14; *p* < 0.01; two quarters), although there was no significant association with levofloxacin resistance (β = 0.23; *p* = 0.35) (Table 3).

**Table 3:** Cross-correlation coefficients between quarterly antibiotics prescriptions and quarterly isolation rates of antibiotic-resistant *K pneumoniae* from 2012 to 2016

Antibiotics	Antibiotic-resistant *Klebsiella pneumoniae*		
	Piperacillin/tazobactam	Ceftazidime	Levofloxacin
β-lactam/β-lactamase inhibitors	0.66 *p* < 0.01 2 quarters lag	0.54 *p* = 0.02 2 quarters lag	−0.60 *p* = 0.01 2 quarters lag
Third-generation cephalosporins	−0.11 *p* = 0.65 -	0.64 *p* < 0.01 2 quarters lag	0.50 *p* = 0.03 1 quarter lag
Fluoroquinolones	0.26 *p* = 0.29 -	0.14 *p* < 0.01 2 quarters lag	0.23 *p* = 0.35 -

## Discussion

The emergence of ESBL-producing *K. pneumoniae* strains and the increasing rates of carbapenem resistance have reduced therapeutic options for infections caused by these organisms [2, 6, 10]. BLBLIs such as piperacillin/tazobactam have been suggested as alternatives to carbapenems [10, 11, 12] and may be comparable in efficacy to carbapenems against ESBL-producing *K. pneumoniae* infections [34]. However, although the evidence for their use as an alternative to carbapenems has been conflicting [12], their use has been increasing. This has led to increasing concern about resistance, which undermines the efficacy of these drugs [12].

To improve policies around the potential replacement of carbapenems with BLBLIs, it is important to examine the relationship between BLBLI use and antimicrobial resistance using a seasonal analysis, which previous studies used to demonstrate the linkage between seasonality and antibiotic use [22, 35].

The greatest positive temporal association was found between all BLBLI utilization and piperacillin/tazobactam resistance in *K. pneumoniae*. However, no significant correlation was found for the use of the third-generation cephalosporins and fluoroquinolone. This was presumably due to the selection pressure of antibiotic use on the prevalence of resistant isolates. This finding is consistent with results of a previous study that identified a significant correlation between piperacillin/tazobactam use and subsequent piperacillin/tazobactam resistance in *Enterobacteriaceae* [36].

We also found that ceftazidime resistance in *K. pneumoniae* temporally correlated with prescriptions for all BLBLIs, all third-generation cephalosporins, and all fluoroquinolones. Consistent with our findings, a previous report indicated that the presence of ceftazidime-resistant *Klebsiella* spp*.* is significantly positively associated with cephalosporin and fluoroquinolone use at the hospital level [37]. Regarding the positive association between fluoroquinolone use and the prevalence of ceftazidime resistance to *K. pneumoniae,* this may be explained by the transferrable plasmid (qnr gene) among ESBL-producing strains which leads to quinolone resistance [38]. Thus, these correlations are likely due to the ability of ESBLs to confer resistance to multiple classes of antibiotics [39].

However, we did not find an association between levofloxacin resistance in *K. pneumoniae* and the consumption of quinolones; this result was in contrast to some previous reports which found that increasing fluoroquinolone use was associated with rising incidence of quinolone resistance in *Enterobacteriaceae* [40, 41]. Additionally, we found that levofloxacin resistance in *K. pneumoniae* was negatively associated with all BLBLI utilization and positively associated with third-generation cephalosporin utilization. These results may be due to the low level of fluctuation from the quarterly number of fluoroquinolones used, and the low magnitude of utilization likely affected the resistance emergence rates [42].

This is the first study to describe the temporal relationship between BLBLI use and the resistance to them in *K. pneumoniae* in a tertiary level hospital. The strong correlations reported in our study suggest that replacing carbapenems with BLBLIs is not an ideal long-term solution, as resistance is likely to spread rapidly. A previous study in Korea showed that broad-spectrum penicillins including BLBLIs were more frequently prescribed than narrow-spectrum penicillins [43]. Another study demonstrated that this is presumably due to the physician’s desire to avoid missing an unlikely or unidentified resistant pathogen [44]. Thus, BLBLIs should be suggested to be used with caution as a carbapenem-sparing option. Further research is needed to evaluate the impact of antimicrobial stewardship programs to balance the use and resistance across drug classes.

Although the results from our study were significant, there are some limitations. First, the study was ecological in nature and may not represent biological associations at the individual level. However, ecological studies are often used to suggest the relationship between use of an antibiotic and resistance to that antibiotic on population level [35, 45]. Second, we used the total number of antibiotics prescriptions as a measure of antibiotic consumption instead of using defined daily doses (DDDs) in this study. However, a previous study indicated that evaluating the total number of antibiotics prescriptions is a useful method to measure the strength of the relationship between antibiotic consumption and antibiotic resistance [41], and DDDs are typically highly correlated with the total number of prescriptions. Third, other factors such as the inconsistency of the patient population, transferred patients having resistant organisms from different hospitals, possible epidemics, and infection control measures during the study the period may have affected the level of resistance in our findings.

## Conclusions

In this study, we identified a significant temporal correlation between BLBLI use and piperacillin/tazobactam resistance in *K. pneumoniae*. The increasing trend in the rates of resistance to piperacillin/tazobactam in *K. pneumoniae* observed in this study raises concerns regarding the appropriate use of BLBLIs as carbapenem-sparing antibiotics. Our results suggest that new interventions aimed at reducing the use of BLBLIs may help retain their efficacy as a carbapenem-sparing alternative.

## Abbreviations

BLBLIs: β-lactam/β-lactamase inhibitors
CLSI: Clinical Laboratory Standards Institute
DDDs: Defined daily doses
ESBLs: Extended-spectrum β-lactamases
KHIRA: Korean Health Insurance Review and Assessment Service
WHO: World Health Organization
